# Recombinant Production of Horseradish Peroxidase Conjugates with Fab Antibodies in Pichia pastoris for Analytical Applications

**Published:** 2011

**Authors:** O.V. Koliasnikov, V.G. Grigorenko, A.M. Egorov, S. Lange, R.D. Schmid

**Affiliations:** Kolmogorov Advanced Education and Science Center, Lomonosov Moscow State University; Department of Chemistry, Lomonosov Moscow State University; Institute of Technical Biochemistry, University of Stuttgart

**Keywords:** horseradish peroxidase, antibodies, recombinant conjugates, *Pichia pastoris*expression

## Abstract

Recombinant immunoconjugates of marker enzymes with antigens or antibodies present considerably more advantages than those obtained by conventional methods of chemical synthesis; i.e. they are homogeneous, have a strictly determined stoichiometry, and retain the functional activity of both a marker protein and an antigen/antibody. Based on the pPICZαB shuttle vector, we first managed to obtain a recombinant conjugate of key marker enzyme horseradish peroxidase (HRP) with*Fab*fragments of antibodies against atrazine. The resulting genetic construction allows us to switch to any other antibody sequence, via the simple re-cloning of variable parts and an additional reporter enzyme. Conjugates were successfully produced in the*Pichia pastoris*methylotrophic yeast expression system. The target activity of the conjugates (both enzymatic and antigen-binding) has been demonstrated by ELISA method.

## INTRODUCTION

Enzyme immunoassays for the detection and quantitative analysis of various substances are based on coupling of marker enzymes such as horseradish peroxidase (HRP, EC 1.11.1.^7^]) with antigens or antibodies. However, all major approaches used for the chemical conjugation of proteins and haptens result in the partial inactivation of the enzyme and conjugate heterogeneity, which affects the specificity and sensitivity of the ELISA. Genetic engineering can be used to obtain recombinant conjugates of proteins with antigens or antibodies. Such conjugates present a number of advantages; firstly, they have a homogenous composition, secondly, they possess 1 : 1 stoichiometry, thirdly they retain the functional activities of both the marker protein and that of the antigen/antibody, in addition to the reproducibility and the fact that they are relatively simple to produce. Recombinant conjugates of antibodies with alkaline phosphatase [1–3], luciferase [4], and peroxidase *Arthromyces ramosus* [5] were obtained earlier.

The recombinant conjugate of HRP with the earlier obtained fatty-acid-binding protein (FABP) [6] was expressed in *Escherichia coli * cells and used as an immunotracer when performing immunoenzyme assay aimed at the early diagnosis of myocardial infarction. 

The functional expression of the recombinant conjugate of HRP and antibody fragments in *E. coli * is associated with a number of difficulties, since there is no post-translational glycosylation of proteins in *E. coli* cells, resulting in low solubility and aggregation of the expressed/obtained protein. This problem can be solved by replacing the expression system. For instance, it has been shown that methylotrophic yeast *Pichia pastoris * is a more suitable organism/system for antibody expression than *E. coli * cells [7, 8].

HRP [9] and antibody fragments [10] were successfully expressed individually in *P. pastoris * cells, both in the single-stranded form scFv [11, 12] and in a Fab form [13]. Moreover, certain immunoconjugates have also been created using this expression system [14–16]. It has been demonstrated that gene expression in the *P. pastoris * system in the secreted form considerably simplifies the scaling of the process for biochemical applications [17].

The recent advance in the functional expression of HRP and antibodies in secreted form paves the way for the construction of recombinant HRP–antibody conjugates to be used in immunoassays. Firstly, we obtained recombinant conjugates of HRP and Fab-fragments of antibodies against atrazine, in order to study the opportunities provided by this approach. In these chimeric proteins, the peroxidase part is combined with the N- and C-terminal parts of the heavy chain of an antibody via a short linker sequence. The universal vectors for the expression of conjugates of HRP and variable chains of Fab fragments of antibodies were obtained (a simple replacement of the variable part of a heavy and light chain of any other antibody by re-cloning at the PstI/BstEII and BamHI/XhoI sites, respectively) in the secreted form in *P. pastoris * cells *. * A functionally active HRP–Fab (atrazine) conjugate was obtained, possessing antigen-binding properties that are similar to those of monoclonal antibodies, which has been attested by single-stage competitive immunoassay of atrazine (IC _50_ ~ 3 ng/ml).

## EXPERIMENTAL

**Reagents**

The reagents were purchased from the companies Sigma, Fluka, and Difco and used without further purification. Protein electrophoresis (SDS-PAGE) was performed according to the standard procedure, using a low molecular weight protein kit (LMW, Bio-Rad) as the molecular weight standards. The preparative work with DNA was performed using a QIA prep Spin Miniprep Kit and a QIAquick Gel extraction Kit (Qiagen, Germany). Enzymes for DNA restriction and modification were purchased from New England Biolabs, Boehringer-Mannheim, GIBCO-BRL-Life technologies, and MBI. Oligonucleotides for sequencing and PCR were purchased from ARK Scientific, MWG Biotech, or Interactiva (Germany).

**Data processing and presentation**

The gene engineering part of the study was planned using CloneManager software (Scientific & Educational Software, Cary, United States). The spatial structures of immunoconjugates were simulated and visualized on the InsightII (BioSym Inc., United States) software package (BioSym Inc., United States) on an SGI R4400 operating station. The experimental data were prepared for publication using software from the OpenOffice.org (www.openoffice.org) and GIMP (GNU Image Manipulation Program) packages.

**Microorganisms, media, plasmids, and oligonucleotides**

*E. coli*strainDH5α was used for genetic manipulations, and *E. coli* strain BL21(DE3) pLysS (Novagen) was used for intermediate production of the protein. The cells were cultured in an LB medium (1% yeast extract, 1% Peptone, 0.5% NaCl) supplemented with 25 mg/l of Zeocin (Invitrogen).

*Preparing competent cells. E. coli *cells were grown overnight in 50 ml of the LB medium until OD _600 _ was 0.4–0.6 and were isolated from the culture medium by centrifugation (3500 rpm, 4 ^о^ С) for 10 min. The cell precipitate/pellet was re-suspended in a TSS buffer (buffer based on a LBS medium containing 10 g of PEG-6000, 5 ml of DMSO, and 0.6 g of MgCl _2_ in 100 ml; рН 6.5) and then kept on ice for 1 h, aliquoted (200 µl), and quickly frozen at –80°С.

Recombinant antibodies and their conjugates with HRP were expressed using *P. pastoris* X33 (Invitrogen) and shuttle vector pPICZαB (Invitrogen) for cloning.

The NotI site was removed using forward and reverse primers ( *Table* ). A three-stage PCR was used (primers listed in the *Table* ), in order to incorporate the *HRP* gene behind the gene of the heavy antibody chain and to remove the restriction sites BspCI, ApaI, PstI, BstEII, BglII, XhoI, BamHI, SacI, and PvuI. 

**DNA modification and cell transformation**

Manipulations with DNA included the standard procedures [18]. *E. coli * cells were transformed via the addition of plasmids or a ligation mixture to the unfrozen competent cells. *P. pastoris * cells were also transformed by plasmids preliminarily linearized at the PmeI site via electroporation.

*P. pastoris* **cultivation and secretion of the recombinant conjugate**

*P. pastoris *cells were cultivated in a YPD medium (1% yeast extract, 2% Peptone, 2% *D* -glucose). The target protein was synthesized in the glucose-free YP medium, using 0.5 vol % methanol as an inducing agent. The YPDS medium(YPD containing 1 M sorbitol) was used for transformation of *P. pastoris * cells *. * The solid medium contained 1.5% of Bacto Agar. The transformants were grown in the YPDS medium at 30°С under stirring (200 rpm) until OD _600 _ = 15 units was obtained. The cells were centrifuged at 3,000  *g * and 4°С, washed with YP medium, and OD _600 _ was brought to 1. The induction was performed for 96 h by adding 0.5 vol % methanol every 24 h. The supernatant was concentrated via membrane ultrafiltration (Amicon, 10 kDa).

**Synthesis of bovine serum albumin (BSA) conjugated with atrazine**

The mixture of 1 mg of atrazine derivative (4-chloro-6-(isopropylamino)-1,3,5-triazine-2-(6-amino-caproic acid)) (~ 3.2 µmol), 1.7 mg of N-hydroxysuccinimide (~ 15 µmol), 6.6 mg of N,N’-dicyclohexylcarbodiimide (~ 30 µmol) in 130 µl of 1,4-dioxane was stirred for 8 h at room temperature. The precipitate was isolated by centrifuging on a desktop centrifuge (12,000 rpm, 30 s). The supernatant was added dropwise to the BSA solution (2 mg) in 3 ml of 0.13 M NaHCO _3_ . The reaction mixture was left in a dark place for 3 h. The reaction product was applied to a PD-10 column that was preliminarily balanced with a phosphate-buffered saline (PBS), pH 7.5. A total of 16 fractions (0.5 ml each) were collected and analyzed spectrophotometrically (at 220 and 260 nm). The fractions with the highest OD _220/260 _ ratio were combined to be used in further experiments.

**Determination of activity of the recombinant conjugate by ELISA**

ELISA was performed overnight using plates (“NUNC” MAXI-SORP) with the preliminarily sorbed BSA-atrazine (1 : 100 dilution) conjugate or BSA (10 µg/ml) in a 10 mM carbonate buffer, pH 9.0, at 4°С. The samples of supernatant of a *P. pastoris * culture medium were successively diluted in PBS, added into the plate wells, and incubated at 37°С for 1 h. Then, the plate was washed thrice with PBS containing 0.1% of Tween-20 (PBS-T), and 50 µl of a TMB substrate mixture was added (0.6 mg/ml TMB and 8 mM H _2_ O _2_ in 0.1 М acetate buffer, pH 5). The reaction was stopped by adding 50 µl of 2 M H _2_ SO _4_ ; the optical density was measured at 450 nm.

**Competitive ELISA for determining atrazine**

150 µl of the calibration sample (0.1, 1.0, 10, 20, 50, 100, 500 ng/ml of atrazine in PBS-T) and 40 µl of a recombinant conjugate solution were placed into plate wells with the preliminarily sorbed BSA-atrazine conjugate and incubated at 37°С for 1 h. The plate was washed three times with PBS-T, and 50 µl of the TMB substrate mixture was added into each well. The reaction was stopped by adding 50 µl of 2 M H _2_ SO _4_ ; the optical density was measured at 450 nm.

## RESULTS AND DISCUSSION

Recombinant conjugates are chimeric proteins combining the structural components of both marker enzymes and an antigen/antibody. The use of modern approaches has almost solved the problem of obtaining such recombinant enzymes as alkaline phosphatase, β-galactosidase, luciferase, and horseradish peroxidase that are used as markers in the ELISA methods. However, the production of recombinant conjugates is an appreciably complicated task, since it remains thus far impossible to reliably predict the structure of the desired conjugate; hence, loss of the functional activity of both the marker enzyme and antigen is possible, due to the incorrect folding of two components.

Recombinant conjugates comprising bacterial enzymes (β-galactosidase and alkaline phosphatase) that can be easily expressed in soluble form in *E. coli* cells, as well as some other enzymes, were earlier obtained. The major problem associated with the use of β-galactosidase and alkaline phosphatase within conjugates is their tetrameric and dimeric structures, respectively, which results in a considerable increase in conjugate affinity in comparison with a free antibody. This phenomenon is particularly undesirable when designing ELISA competitive schemes. Meanwhile, horseradish peroxidase, one of the marker enzymes that have been most widely used in ELISA, is expressed in *E. coli * cells only in the form of inclusion bodies, which until recently has impeded the obtaining of an active enzyme.

The recent advance in the heterologous expression of antibody genes in the cells of methylotrophic yeast *P. pastoris * offers great opportunities for using this system for the synthesis of the conjugates of horseradish conjugates with antibodies in the secreted soluble and functionally active forms.

**Designing the expression vector to obtain a recombinant conjugate of HRP with a Fab fragment of antibodies in**

*P. pastoris*

The expression system for obtaining recombinant conjugates of HRP and Fab fragments of antibodies was elaborated on the basis of the pPICZαB vector. The genetic construction was placed under the control of the AOX promoter containing the PmeI site for the subsequent linearization and recombination into the yeast genome. The vector also contains the signal sequence of α-factor, which is necessary for the directed secretion of recombinant protein into the culture medium. Gene s *h ble * provides zeocin resistance of both *E. coli* and * P. pastoris * cell types. The possibility of introducing a hexahistidine sequence at the C-terminus of the recombinant protein is provided to simplify the procedures of extraction and purification of the product.

**Fig. 1:**
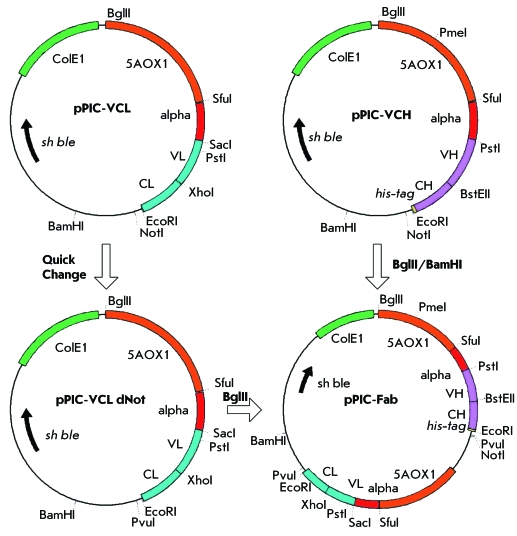
Cloning scheme for construction of pPIC Fabplasmid.

We used the plasmids earlier obtained (pPIC-VCL and pPIC-VCH [19] containing the corresponding fragments of variable regions of the light and heavy chains of K4E7 monoclonal antibody against atrazine [20], respectively) as the starting material ( *Fig. 1* ). Both these vectors contained the NotI site behind the cloned gene.

To design the universal construction, we planned to leave only one NotI site in the vector behind the gene of the antibody heavy chain. PCR (the QuickChange mode [21]) using a special primer pair ( *Table* ) was employed to remove the NotI site from the pPIC-VCL plasmid. The vector obtained by this procedure is known as pPIC-VCL dNot. Then, the BglII/BamHI fragment of the pPIC-VCH plasmid containing the heavy-chain gene was cloned at the BglII site of plasmid pPIC-VCL dNot prior to the light chain gene. The expression vector pPIC-Fab was used. The cloning scheme is given in *Fig. 1.*

**Table 1:** Forward (F) and reversed (R) primers used for generation of genetic constructs with PCR. The introduced mutations are indicated in bold and underlined in primer sequences

Target gene	Restriction site destroyed		Primer sequence*
HRP	NotI	R	5’-CGATCGAGCC GCGATGGCCG CCAGC-3’
HRP	NotI	F	5’-GCTGGCGGCC ATCGCGGCTC GATCG-3’
Fab VCH		R	5’-AGGCACAGCT ATAGGTACG-3’
Fab VCH		F	5’-TGAGAACCTC CACCGCCGCA GTCGCGCGGT ACG-3’
HRP		R	5’-GCGGCGGTGG AGGTTCTCAG TTAACGCCGA CTTTCTACG-3’
HRP	PstI, BspCI	R	5’-GACCGCATGA AGGCTGCTGT CG-3’
HRP	PstI, BspCI	F	5’-ACAGCAGCCT TCATGCGGTC G-3’
HRP	BstEII, ApaI	R	5’-ACTCTAGCCG GCGGTCCCTC-3’
HRP	BstEII, ApaI	F	5’-GGACCGCCGG CTAGAGTGAC-3’
HRP	XhoI	R	5’-GAACCGTTCG AGTGATCTAG-3’
HRP	XhoI	F	5’-AGATCACTCG AACGGTTCAG-3’
HRP	SacI	R	5’-GATCAGGAGC TGTTCTCATC-3’
HRP	SacI	F	5’-AACAGCTCCT GATCAGATTG-3’
Fab VCL	NotI	R	5’-ATCGGTACCT CGATCGAGCC GCGATGG-3’
Fab VCL	NotI	F	5’-TGAAGTGGTA CGGCGATGC-3’

The nucleotide sequence regions that were changed are highlighted.

**Fig. 2:**
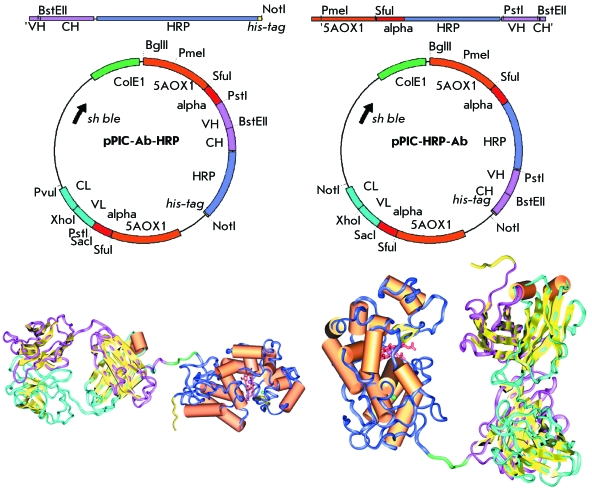
General versatile expression vectors for recombinant conjugates of *HRP* with Fab antibody fragments production. The spatial models of recombinant conjugates of Fab-HRP and HRP-Fab are shown in the left and right panels, respectively.

The universal pPIC-Fab gene obtained contains SacI/XhoI and PstI/BstEII site pairs for simple cloning of the genes of the heavy and light chains, encodes the C-terminal hexahistidine fragment for simplifying the purification of the target protein using metal chelate chromatography, and the NotI site for cloning the marker protein (such as HRP, green fluorescent protein (EGFP), luciferase, etc.) at the C-terminus of the heavy chain of the antibody.

A vector for the expression of the recombinant conjugate of peroxidase with Fab fragments of antibodies was designed simultaneously. For the simplicity of cloning, restriction sites PstI, BstEII, BglII, XhoI, SacI, PvuI *, * ApaI, BamHI and BspCI were removed using the primers listed earlier ( *Table* ) from the initial *HRP* gene [22] that was preliminarily cloned in the corresponding pPIC vector. Either before the *HRP * gene or behind it the fragments of antibody genes were simultaneously cloned. Thus, three-stage PCR was used to obtain two genetic constructions in which the *HRP* gene was linked with the sequence encoding the N-terminal region of the variable part of the heavy Fab chain or the C-terminal region of the constant part of the heavy chain via a short linker sequence (Gly _4_ Ser) _3 _ ( *Fig. 2* ). It should be mentioned that in order to avoid the formation of nonfunctional dimers of the light chain, genetic constructions were cloned in the pPIC-Fab vector at the sites PmeI/BstEII and BstEII/NotI, respectively; the heavy chain was selected for the cloning of the marker protein gene. The mutual arrangement of genes in plasmids pPIC-Ab-HRP and pPIC-HRP-Ab was confirmed by restriction analysis and sequencing.

**Expression and purification of recombinant conjugates Fab-HRP and HRP-Fab**

**Fig. 3:**
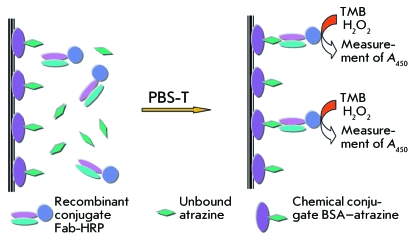
ELISA scheme for atrazine determination.

**Fig. 4:**
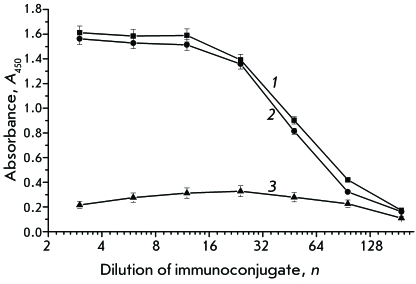
Titration of recombinant conjugates: 1 – Fab-HRP (clone 1.1); 2 – Fab-HRP (clone 1.2); 3 – HRP-Fab (clone 8).

*P. pastoris* X33 cells (Invitrogen) were transformed via the plasmid vectors pPIC-Ab-HRP and pPIC-HRP-Ab using electroporation with an efficiency of approximately 100 clones per 10 µg of plasmid DNA. The expression of the target protein was tracked by increasing the peroxidase activity in the supernatant of the culture medium, reaching a plateau on day 5 of cultivation. Ten clones were analyzed with each construction. The HRP activity with respect to the TMB substrate was detected only in three clones out of 20: two clones (1.1, 1.2) corresponding to pPIC-Ab-HRP and one clone (8) corresponding to pPIC-HRP-Ab. These clones were selected for further consideration.

As shown by SDS-PAGE electrophoresis (the data are not provided), the blurred bands located below the band at 100 kDa correspond to the recombinant conjugates HRP-VCH and VCH-HRP. This blurriness of the bands is accounted for by the microheterogeneity of conjugates, conditioned by excessive glycosylation that is typical for *P. pastoris* ; correlating with our data and the data published on the expression of the *HRP* gene [9]. A considerably greater excess (by a factor of 3–4) of light-chain molecules was observed (the band at 25 kDa) in comparison with that found during the expression of the Fab fragment [19]. Unexpectedly, it occurred that the recombinant conjugated manifested no enzymatic activity toward another peroxidase substrate, ABTS, as opposed to TMB. It is well known that the site of binding with ABTS is located in the hydrophobic region on the HRP surface, in the so-called “Phe patch” zone [25]. This zone is noticeably distant from the active site of HRP, and it can be assumed that the substrate binding to it is complicated due to steric reasons – as a result of excessive glycosylation or the presence of a Fab fragment of antibody. The first hypothesis is more probable, since the same effect is observed under both positions of the heavy chain of the antibody with respect to HRP. Moreover, a similar effect was earlier observed upon expression of the *HRP* gene in *P. pastoris* (the data have not been published).

The total yield of recombinant conjugates was approximately 3–10 mg per 1 l of the *P. pastoris* culture supernatant. A relatively low yield of secreted conjugates correlates with the yield upon expression of the *HRP* gene only. We believe that one of the factors that have a negative effect on the yield of the secreted product is the excessive glycosylation of the peroxidase component of the conjugate, which is typical of *P. pastoris* cells. In order to verify this hypothesis, it may be reasonable to remove all N-glycosylation sites in HRP or replace HRP with another reporter protein, such as EGFP.

**Characterization of recombinant conjugates by ELISA**

**Fig. 5:**
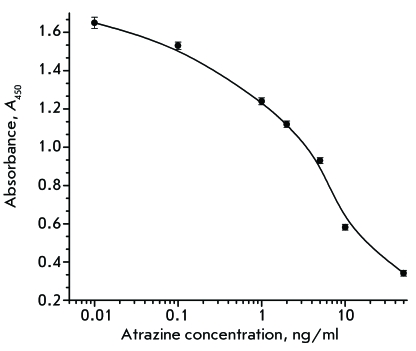
Calibration curve for atrazine determination in competitive ELISA with recombinant conjugate of Fab-HRP (clone 1.1).

In order to confirm the antigen-binding activity of recombinant conjugates, we selected the scheme of indirect competitive single-stage ELISA ( *Fig. 3* ) carried out on the wells with an immobilized atrazine–BSA conjugate. The binding of recombinant conjugates to atrazine was preliminarily studied ( *Fig. 4* ). The data obtained attest to the presence of both catalytic and antibody activity in all three clones. However, the low activity of the HRP-Fab sample (clone 8) in comparison with the C-terminal conjugate Fab-HRP (clones 1.1 and 1.2) may attest to the fact that the mutual spatial arrangement of two components of the chimeric protein in this case results in a decrease in the catalytic activity of peroxidase. The samples of recombinant conjugates Fab–HRP (clones 1.1 and 1.2) have similar characteristics, and specimen 1.1 was used for further ELISA determination of atrazine. The typical calibration diagram ( *Fig. 5* ) allows one to determine the atrazine concentration over a wide range, from 0.1 to 50 ng/ml; the variation coefficient being no higher than 8%. IC _50 _ is equal to 3 ng/ml, which agrees well with the results of atrazine determination by a two-stage ELISA procedure using recombinant Fab fragments of the same antibody K411B [19] and with the data on the single-chain mini-antibody (scFv) obtained earlier in *E. coli* [24]. Meanwhile, in the initial monoclonal antibody, the IC _50 _ value was equal to 0.2 ng/ml [19]. As is evident in the majority of similar cases, the fact that the IC _50 _ value differs from that of recombinant antibodies is in all likelihood connected with the bivalence of the initial monoclonal antibody.

Thus, the recombinant conjugates of peroxidase with Fab fragments of antibody against atrazine obtained in the present study possess functional activity and can be used to determine atrazine via ELISA.

## CONCLUSIONS

The possibility of using a recombinant, functionally active HRP (as a marker enzyme) conjugated with Fab fragments of the antibody against atrazine was shown for the first time. In the present study, recombinant conjugates were obtained in which the Fab fragment of an antibody is bound both to the N- and the C- terminuses of the marker enzyme. Both these variants manifest immunological and catalytic activity.

The functional secretion of recombinant conjugates of HRP with Fab fragments of antibodies offers opportunities for broad application in ELISA. The results obtained will be used to design highly sensitive immunobiosensors of a new generation, based on the recombinant DNA technology. 

## Abbreviations

HRP: horseradish peroxidase
ELISA: enzyme-linked immunosorbent assay
BSA: bovine serum albumin
PCR: polymerase chain reaction
TMB: 3,3’,5,5’-tetramethylbenzidine
ABTS: 2,2’-azinobis(3-ethylbenzothiazoline-6-sulphonic acid)
